# The dynamic balance of the skin microbiome across the lifespan

**DOI:** 10.1042/BST20220216

**Published:** 2023-01-06

**Authors:** Elizabeth C. Townsend, Lindsay R. Kalan

**Affiliations:** 1 Department of Medical Microbiology and Immunology, School of Medicine and Public Health, University of Wisconsin-Madison, Madison, U.S.A.; 2 Microbiology Doctoral Training Program, University of Wisconsin-Madison, Madison, U.S.A.; 3 Medical Scientist Training Program, School of Medicine and Public Health, University of Wisconsin-Madison, Madison, U.S.A.; 4 Department of Medicine, Division of Infectious Disease, School of Medicine and Public Health, University of Wisconsin-Madison, Madison, U.S.A.; 5 M.G. DeGroote Institute for Infectious Disease Research, David Braley Centre for Antibiotic Discovery, Department of Biochemistry and Biomedical Sciences, McMaster University, Hamilton, Ontario, Canada

**Keywords:** host–microbe interactions, microbiome, skin

## Abstract

For decades research has centered on identifying the ideal balanced skin microbiome that prevents disease and on developing therapeutics to foster this balance. However, this single idealized balance may not exist. The skin microbiome changes across the lifespan. This is reflected in the dynamic shifts of the skin microbiome's diverse, inter-connected community of microorganisms with age. While there are core skin microbial taxa, the precise community composition for any individual person is determined by local skin physiology, genetics, microbe–host interactions, and microbe–microbe interactions. As a key interface with the environment, the skin surface and its appendages are also constantly exchanging microbes with close personal contacts and the environment. Hormone fluctuations and immune system maturation also drive age-dependent changes in skin physiology that support different microbial community structures over time. Here, we review recent insights into the factors that shape the skin microbiome throughout life. Collectively, the works summarized within this review highlight how, depending on where we are in lifespan, our skin supports robust microbial communities, while still maintaining microbial features unique to us. This review will also highlight how disruptions to this dynamic microbial balance can influence risk for dermatological diseases as well as impact lifelong health.

## Introduction

The skin harbors complex microecosystems of bacteria, fungi, and viruses, each with distinct adaptions to survive on the skin. However, there is no single definition of a balanced skin microbiome. For any individual the microbial balance is dynamic, maturing with us as we grow and navigate through the environments around us. Across our lifespan, the stability and function of skin microbial communities are driven both by interactions with the host and between microorganisms.

Human skin varies in its physical characteristics across body sites [1–4], ranging from oily / sebaceous to moist or dry, resulting in distinct microenvironments that promote unique microbial populations. Sebaceous sites (e.g. face, chest, and back) have a high density of hair follicles and sebaceous glands. The lipid rich sebum produced by these glands promotes colonization by lipophilic taxa, primarily *Cutibacterium* bacteria and *Malassezia* fungi [4–6]. Moist sites (e.g. elbow crease, axilla, and groin) have high concentrations of apocrine sweat glands and are dominated by Staphylococc*us* spp. and *Corynebacterium* spp*.*[4]. In contrast with other sites, dry sites (e.g. forearm, abdomen, and palms) have the lowest abundance yet greatest microbial diversity, with significant populations of *Cutibacterium, Corynebacterium,* and *Streptococcus* species [2]. Thus, at a microenvironmental level, the balance of skin microbial communities is partially dictated by these physiologic and abiotic features of the skin niche.

The skin is an immunologically rich organ. To maintain the skin barrier tissue-resident immune cells collectively sample and respond to microbial products [4], produce antimicrobial peptides (AMPs) [7], and ultimately prevent penetration of skin microbes into deeper skin layers or open wounds. A hallmark of a healthy equilibrium among skin microbial communities is the maintenance of skin barrier integrity. This is accomplished by promoting skin cell maturation [8,9], training the immune system [10–12], and preventing pathogen overgrowth through niche exclusion and competitive interactions such as the production of antimicrobials [13–16]. Disruption of this equilibrium is characterized by the overgrowth of some bacterial species, such as *Staphylococcus aureus*, and an overall loss of community diversity [2]. This dysbiosis can lead to impaired wound healing, increased inflammation, and greater risk for infection [2,17–19].

Finally, microbe–microbe interactions within the skin microbiome can drive overall community structure. The three prominent skin taxa, *Cutibacterium acnes, Corynebacterium* spp., and coagulase-negative staphylococci (CoNS) are known to mediate other microbial taxa in the microenvironment and have been extensively reviewed elsewhere [5,20]. Key interactions and recent studies are summarized in Table 1.

**Table 1:** Key microbe–microbe interactions on the skin

Microbe Producing the Molecule	Anti-microbial molecule (Type)	Inhibited Microbes	Proposed Mechanism	Source
*Cutibacterium acnes*	Acnecin (peptide)	non-anecin producing *C. acnes*	Exact mechanism Unknown. Proposed to help *C. acnes* phylotypes maintain dominance within a pore.	[105,164]
		*Corynebacterium*		
	Cutimycin (thiopeptide)	MRSA	Unknown	[14]
		*Staphylococcus epidermidis*		
	Propionic acid (SCFA)	MRSA	Lowers local pH. This can limit the growth of several pathogens while not significantly affecting growth of skin commensals.	[165-168]
		*Escherichia coli*		
		*Candida albicans*		
	Propionic acid (SCFA)	*Staphylococcus epidermidis*	Inhibit bacterial biofilm formation. These SCFA also influence melanocyte, keratinocyte, and sebocyte gene expression, and modulate host inflammation.	[114, 169-173]
	Isobutyric acid (SCFA)			
	Isovaleric acid (SCFA)			
*Corynebacterium accolens*	Unidentified protein	*Staphylococcus aureus*	Inhibits biofilm formation	[13]
		MRSA		
	LipS1 (lipase)	*Streptococcus pneumoniae*	Metabolizes host lipids into free fatty acids that inhibit bacterial growth	[174,175]
	Unidentified secreted protein(s)	*Staphylococcus aureus*	Inhibits the agr quorum sensing system and prevents agr-dependent virulence factor expression	[176]
*Corynebacterium amycolatum*	Unidentified secreted protein(s)	*Staphylococcus aureus*	Inhibits the agr quorum sensing system and prevents agr-dependent virulence factor expression	[176]
*Corynebacterium striatum*	Unidentified secreted protein(s)	*Staphylococcus aureus*	Inhibits the agr quorum sensing system and prevents agr-dependent virulence factor expression	[176]
*Corynebacterium pseudodiptheriticum*	Unidentified secreted protein(s)	*Staphylococcus aureus*	Inhibits the agr quorum sensing system and prevents agr-dependent virulence factor expression	[176]
	Unidentified secreted factor	*Staphylococcus aureus*	Bactericidal against *S. aureus* when it expresses agr-dependent virulence factors	[177]
		*MRSA*		
*Staphylococcus capitus*	Capidermicin (AMP)	*Lactococcus lactis*	Forms pores in membranes	[178]
		*Micrococcus leuteus*		
		*Staphylococcus aureus*		
		*Staphylococcus intermedius*		
		*Staphylococcus pseudointermedis*		
	Unidentified bacteriocin(s)	Listeria monocytogenes	Unknown	[16]
		*Staphylococcus aureus*		
		*MRSA*		
		Streptococcus alagactiae		
		*Streptococcus Bovis*		
	PSM-beta 1 to PSM-beta 6	*Micrococcus leuteus*	Induces cell lysis	[179]
	PSM 1 to PSM 4	*Cutibacterium acnes*	Act synergistically for targeted killing	[180]
*Staphylococcus caprae*	Unidentified autoinducing peptide	*Staphylococcus aureus*	Inhibits the agr quorum sensing system and prevents agr-dependent virulence factor expression	[181]
		*MRSA*		
*Staphylococcus epidermidis*	Esp (serine protease)	*Staphylococcus aureus*	Inhibits biofilm formation and destructs biofilms	[182]
	Unidentified AMP	*Staphylococcus aureus*	Targeted killing	[183]
*Staphylococcus hominis*	Unidentified AMP	*Staphylococcus aureus*	Targeted killing	[183]
	Hominicin (AMP)	VRSA	Unknown	[184]
	Unidentified bacteriocin(s)	Listeria monocytogenes	Unknown	[16]
		Streptococcus alagactiae		
		*Streptococcus Bovis*		
Staphylococcus mutans	Mutacin 1140 (AMP)	VRSA	Unknown	[184]
Staphylococcus lugdunensis	Lugdunin (thiazolidine-containing cyclic peptide, AMP)	VRSA	Unknown	[184]
	Lugdunin (thiazolidine-containing cyclic peptide, AMP)	*Enterococcus faecium*	Direct killing	[185]
		Enterococcus faecalis		
		Listeria Monocytogenes		
		*Staphylococcus aureus*		
		MRSA		
		Streptococcus pneumoniae		
	Lugdunin (thiazolidine-containing cyclic peptide, AMP)	Staphylococcus aureus	Direct killing and amplification of innate immune responses	[186]
*Staphylococcus simulans*	AIP-I to AIP-III	*Staphylococcus aureus & MRSA*	Inhibits the agr quorum sensing system and prevents agr-dependent virulence factor expression	[187]
*Staphylococcus warneri*	AIP-I to AIP-II	*Staphylococcus aureus & MRSA*	Inhibits the agr quorum sensing system and prevents agr-dependent virulence factor expression	[188]
	Unidentified bacteriocin(s)	*Staphylococcus aureus*	Unknown	[16]
		Streptococcus alagactiae		

Summary table of prominent and recently identified antimicrobial molecules produced by major skin bacterial taxa to inhibit other microbial community members and/or potential pathogens. The table is organized by the taxa that produces the antimicrobial molecule(s). Where possible the molecule's proposed mechanism of action is included.

Abbreviations: AIP, Autoinducing peptide; AMP, Antimicrobial peptide; MRSA, Methicillin resistant *Staphylococcus aureus*; MSSA, Methicillin sensitive *Staphylococcus aureus*; PSM, phenol-soluble modulins; SCFA, Short chain fatty acids; VRSA, Vancomycin resistant *Staphylococcus aureus.*

## Changes with age

**Figure 1.**
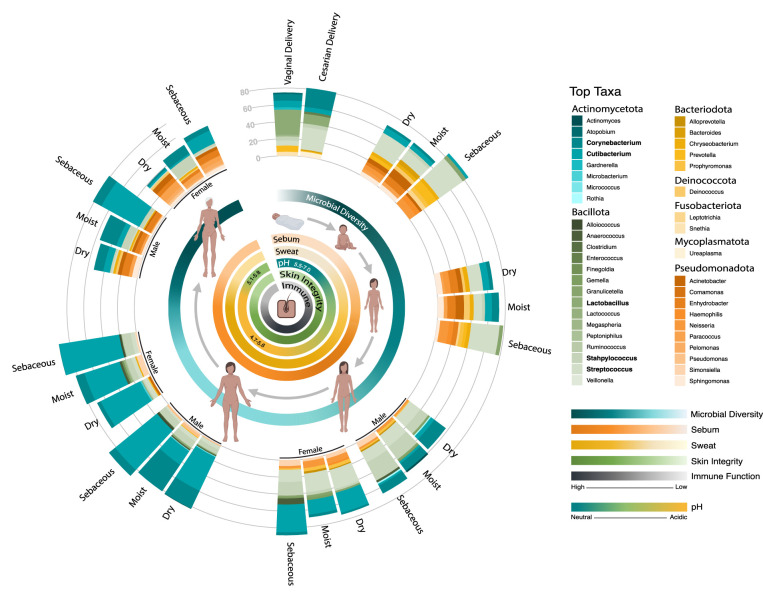
The dynamic balance of the skin and its microbiome over the lifespan. Over a lifetime the skin's physiology changes as an individual's cutaneous immune systems matures and hormones drive sweat and sebum gland development. These changes are associated with shifts in the relative abundance of prominent skin microbial taxa and shifts in the overall microbial community diversity. Microbiome data displays the average relative abundance of the top ten microbial taxa for each group as assessed by high-throughput sequencing of the bacterial 16S ribosomal RNA gene. Taxa with a relative abundance >20% in at least one group are bolded. Groups include newborns born either through vaginal delivery or cesarian section [22] as well as dry, moist, and sebaceous sites for infants (1 year old) [40], children (5 years old) [40], adolescents (Tanner Stage III) [46], adults (20–40 years old) [3], and the elderly (60 and older) [137]. Since sexual differences in skin microbial composition become more pronounced over the course of puberty [46], relative abundance plots for adolescent, adult, and elderly males and females are displayed. Inner circles represent relative microbial diversity, sebum production, sweat production, surface pH, skin integrity, and immune function throughout life [33,46,87,144,152,153].

Homeostatic skin microbial community structures shift as we age and navigate the environment (Figure 1). Throughout a lifetime the skin's physiology changes as the cutaneous immune systems matures and hormones drive sweat and sebum gland development, which impacts the availability of key nutrients. As a direct interface with the environment, the skin also continuously shares microbes with the places and people around us. Below we summarize the shifts in the skin microbiome over the human lifespan and highlight where disruptions to the skin microbiome at critical age-associated stages influence the risk for disease development.

### Birth

The skin's first substantial introduction to microbes occurs at birth, where the skin is quickly colonized from the immediate environment. Mode of delivery has been shown to influence the initial community composition [21–23]*.* For example, the skin microbiome of vaginally delivered newborns is dominated by vaginal-associated flora, primarily *Lactobacillus* and *Prevotella*, and contains a higher abundance of *Candida albicans* [21,22,24]*.* Newborns delivered via cesarean section have microbiomes containing maternal skin-associated microbes, including *Staphylococcus, Streptococcus, Corynebacterium,* and *Cutibacterium.* Cesarian-delivered newborns intentionally exposed to their mother's vaginal flora at birth have microbiomes containing both skin- and vaginal-associated flora [25]. Although these initial communities are transient [22], the order and timing of species colonization influences how strains subsequently interact with one another [26,27]. These priority effects can shape future community structures and have long term implications for the skin, its microbiome, and overall health.

### Infancy and childhood: initial microbial exposures

Skin microbial compositions gradually shift throughout infancy and childhood [22]. These communities are shaped by the skin's functional maturation, the microbiomes of close care givers, and their environment [28–30]. Particularly in infancy initial microbial exposures prime immune development [26,31] and strengthen the skin barrier through promoting proper keratinocyte differentiation and epidermal repair [8].

Newborn and infant skin has a greater water content, higher pH, suppressed sebum production, faster epidermal turnover, and greater antimicrobial properties [32,33]. Within hours after birth the skin surface begins to acidify [29] and initially homogenous microbial communities begin to diverge into body site-specific communities [26,34]. Continued site-specific skin maturation promotes reorganization of the infant microbiota across body habitats over the first few months [22]. Within 3–6 months, associations between microbial taxa and skin metabolic function (e.g. lipid production and pH) are established [35]. Compared with adults, reduced sebum production in early life is associated with lower *Corynebacterium*, *Cutibacterium* and *Malassezia* abundance*,* increased staphylococci and streptococci*,* and a mycobiome dominated by *Candida* species [22,24,34,36–39]. As children age, skin further acidifies and produces more sebum lipids, which promotes a gradual decline in acid-sensitive streptococci and increase in overall community diversity [32,33,40].

Commensal colonization also stimulates immune cell maturation, particularly regulatory T-cell (Treg) localization to developing hair follicles shortly after birth [41–43]. Specific training of commensal antigen-specific Tregs is required for establishing immune tolerance [41] that prevents inflammation against this commensal later in life [10]. However early exposure does not guarantee future tolerance. The simultaneous recognition of multiple pathogen-associated molecules and toxins (e.g. *S. aureus* alpha*-*toxin) during early colonization events limits pathogen*-*specific Treg formation [44]. Without microbial-specific Tregs, *S. aureus* exposures in later life leads to inflammation.

In early infancy, the skin microbiome of close caregivers further contributes to shaping the infant skin microbiome. At 6 weeks of age, infant and maternal skin microbiomes display very similar community structures [22], and throughout childhood the skin will continue to harbor distinct microbial taxa from caregivers [22,40,45]. However with age, older infants have greater skin microbial diversity and more microbes derived from their rural or urban environments [30] and the similarity between a mother and infant microbiome gradually declines [30,45]. Cutaneous fungal populations are also more diverse in childhood with greater interpersonal variation [37,46,47]. These early life variations and interventions in environmental microbial exposures influence the establishment of the skin microbiome and can modulate lifelong immune responses [48–51]. Disruption to the establishment of this equilibrium [26,52] is associated with greater inflammation and can increase a child's risk for atopic dermatitis and allergy development.

### Microbial imbalance in pediatric atopic dermatitis

Atopic dermatitis (eczema; AD) is an inflammatory skin disorder, characterized by dry, inflamed, itchy skin patches. The typical age of onset is between 3–6 months old and it affects roughly 20% of infants and children, 15% of adolescents, and 10% of adults [53,54]. This early age of onset and evidence of skin microbiome dysbiosis preceding AD onset [52], underscore how early life microbial exposures may influence AD risk. Additionally, many children grow out of AD and experience partial or full resolution of symptoms as they enter adolescence or adulthood [54]. This showcases the important influence of the maturation of skin microbiome in AD's natural course.

Skin barrier dysfunction and immune imbalance contribute to AD's pathogenesis. In AD patients, both affected and non-affected areas of skin display increased permeability [55], reduced water retention, high pH [56], and altered lipid composition [57–59]. Subsequently external microorganisms, particularly *Staphylococcus aureus,* can penetrate deeper skin layers. Stressed keratinocytes and microbial antigens trigger type-2 helper T-cell-driven immune responses [60,61], which further exacerbates barrier defects through down-regulation of filaggrin [62,63], disruption of tight junctions, and reduction in stratum corneum lipids [59]. High levels of type-2 cytokines also reduce antimicrobial peptide production, further increasing the skin's susceptivity to *S. aureus* colonization [63]. Thus, a perpetual cycle of poor barrier function, increased microbial and irritant penetration into deeper skin layers, and increased inflammation then itching and further skin damage is developed.

Elevated *S. aureus* abundance can precede AD development [26,52] and several *S. aureus* virulence factors propagate AD related epidermal damage and inflammation [64–66]. Additional microbiome changes in AD include a reduction in *Cutibacterium acnes*, *Corynebacterium, Dermacoccus, Micrococcus,* and CoNS and an increase in *Streptococcus* and some *Malassezia* species [58,61,67–72]*.* These microbial shifts appear to be temporal, with a loss of community diversity and greater *S. aureus* dominance preceding and during AD flares followed by a gradual return to baseline following resolution [73,74]. Finally, early colonization with commensal CoNS (e.g. *S. epidermidis* and *S. hominis)* [26] and Gram-negative bacteria reduces the risk of developing AD [75].

Current AD treatment revolves around eliminating exacerbating factors (e.g. contact with allergens and strong soaps), maintaining skin moisture with emollients and reducing skin inflammation through topical corticosteroids or calcineurin inhibiters [54,76]. Many of these interventions effectively resolve the skin dysbiosis [68,73,74,77–79]. Investigations into potential therapies are increasingly centered around modulating the microbiome with oral prebiotics, probiotics and symbiotics [80,81], topical emollients containing probiotics [82], or microbial transplantation with commensal skin bacteria [75,83–85]. One promising avenue is harnessing CoNS anti-*S. aureus* activity. The transplantation of *S. epidermidis* and *S. hominis* can diminish *S. aureus* virulence, prevent epithelial damage, and reduce inflammation in murine models and clinical trials of atopic dermatitis (AD) patients [84,86].

### Adolescence and puberty: hormonally driven changes

**Figure 2.**
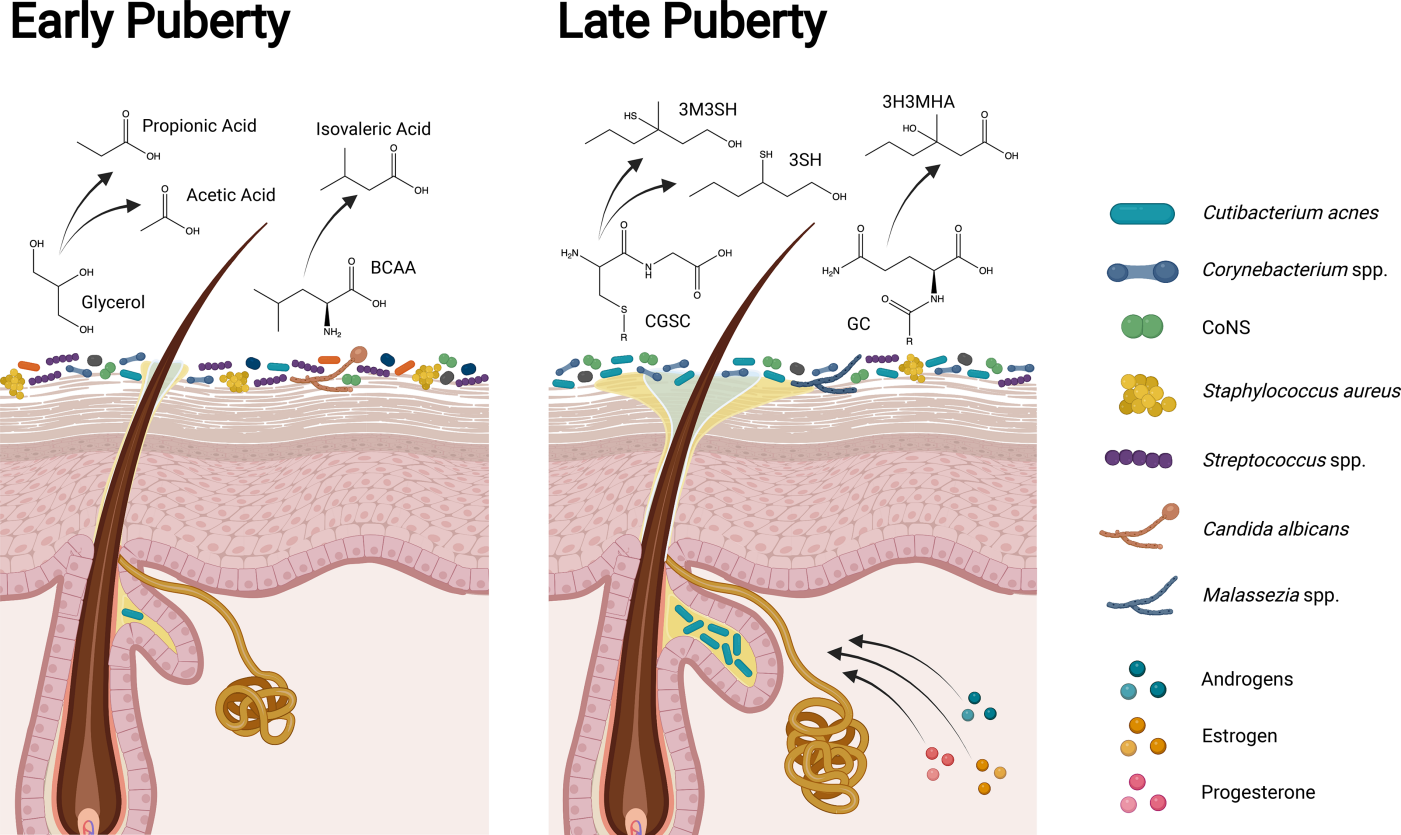
Differences in the skin, microbiome, and body odor production in early and late puberty. In childhood and early puberty (Tanner Stages I to II) the skin microbiome is highly diverse and body odor is associated with CoNS (e.g., *S. epidermidis* and *S. hominis*) production of volatile fatty acids (e.g., propionic, acetic, and isovaleric acid; sour odors) and sulfur (rotten-egg odor) [92]. As puberty advances, steroid hormones promote sebaceous and apocrine sweat gland development [90,91], modify the types of lipids present in sebum [90,97], and enhance the skin barrier [94–96]. In later puberty (Tanner Stages IV to V), increased lipid production and altered lipid content is associated with a skin microbiome dominated by lipophilic taxa [46]. While breakdown of sweat and sebum components into volatile fatty acids still occurs, body odor in young adults becomes more associated with Corynebacterium spp. metabolism of sebum and sweat components into sulfanylalkanols (e.g., 3SH and 3M3SH; oniony odors), and volatile organic compounds (e.g., 3H3MHA; cumin like odors) [92,98–101]. BCAA: Branched chain amino acids; CGSC: Cystine-Glycine-S-conjugate; CoNS: Coagulase negative Staphylococcus spp.; GC: Glutaminyl-conjugate; 3H3MHA: 3-hydroxy-3-methylhexanoic acid; 3M3SH: 3-methyl-3-sullanylhexanol; 3SH: 3-sulfanylhexanol.

Puberty marks the next major shift in our skin microbial communities. The hormones that drive physical and sexual development also directly promote structural and functional changes in the skin such as sebum and apocrine sweat production. This leads to subsequent shifts in microbial composition (Figure 2) [87]. Due to interpersonal variability in puberty onset and progression, the Tanner staging system measures the degree of sexual maturation [88,89]. Tanner stage I corresponds to the pre-pubertal stage and stage V corresponds to adult sexual characteristics. Both cross-sectional and longitudinal studies demonstrate clear shifts in skin microbiome composition across Tanner stages [36,37,46]. Children at stage I have higher relative abundances of *Streptococcus,* Bacteroidota and Pseudomonadota, as well as higher bacterial and fungal diversity compared with young adults at stage V [36,46]. Furthermore, the young adult skin microbiome is dominated by lipophilic microbes, including *Corynebacterium, Cutibacterium acnes,* and *Malassezia* [36,37,46], a composition associated with higher sebum production and serum concentrations of hormones that promote sebum production [46].

Puberty is driven by androgens (e.g. dehydroepiandrosterone and testosterone), estrogen, and progesterone [87]. On the skin androgens promote pubic and axillary hair growth and sex specific hair patterns [87], sebaceous gland development and increased sebum production [90,91], and apocrine sweat gland development with a subsequent increase in body odor [92,93]. Estrogen and progesterone enhance the skin barrier and promote wound healing through encouraging collagen synthesis and stimulating keratinocyte proliferation [94–96]. Puberty-driving hormones also modulate immune function in both pro- and anti-inflammatory ways [96]. These hormonally driven changes in skin physiology lead to changes in the skin microbial communities [46]. For instance, androgen-driven sebaceous gland development [91] and estrogen modification of the lipid types at the skin surface [97] promote the survival of lipophilic species, particularly *C. acnes* and *Malassezia restricta* [46].

Although apocrine gland secretions are initially odorless, microbial metabolism of their components (e.g. branched chain amino acids, fatty acids, and glycerol) produces odoriferous compounds (Figure 2) [92,98]. Body odor in children and young-teens is associated with microbial production of isovaleric and acetic acid (sour odor) and sulfur (rotten-egg odor) [92]. In young adults body odor, particularly from the axilla [92,98,99], is associated with corynebacterial breakdown of sebum into volatile fatty acids (cheesy odor) and sulfanylalkanols (oniony odors) [99–101].

While all puberty-driving hormones surge in both sexes, the heightened role of androgens in males and estrogens plus progesterone in females foster sexual differences in maturation. As such, sexual differences in skin microbial composition become more pronounced as puberty advances [46]*.* Microbial communities of females become less diverse and have greater prevalence of *Cutibacterium* with increasing Tanner stage [46]. Skin microbiomes of males display more inter-individual differences and greater diversity, with higher relative abundance of *Corynebacterium, Staphylococcus, Streptococcus*, and *Haemophilus* [46]. Other hormonally dependent skin physiological (lower pH, increased skin thickness) and immunological changes during puberty likely also account for many maturation-associated skin microbial shifts, but their influence is less defined [36,46].

Collectively, puberty marks major shifts in the skin microbial community structure, metabolic complexity, and function. Microbiome imbalance during this process can promote several microbe-associated skin disorders including acne vulgaris.

### Cutibacterium acnes phylotype imbalance in acne vulgaris

Several microbe-associated skin disorders often begin during puberty, including acne vulgaris hidradenitis suppurativa, and psoriasis, highlighting the influence of puberty-driven skin and microbial shifts in disease pathogenesis [46]. Here we highlight acne vulgaris (acne), due to the strong support for a microbiome-driven association. Although there are microbial disturbances associated with hidradenitis suppurativa and psoriasis, no single microbe or consistent pattern of dysbiosis has been implicated [61,102,103]. Further research into the roles of the microbiome in these conditions are needed.

Acne affects ∼85% of adolescents and young adults (12–25 years old) [104]. Mild acne is characterized by clogged hair follicles while severe cases present with inflammatory, painful papules, pustules, nodules or cysts. Like how individuals experience resolution of AD as they enter adolescence, acne tends to resolve as individuals mature out of adolescence and enter adulthood. This further highlights how the natural age associated evolution of microbial community structures influence the course of a disease.

Our understanding of acne's pathogenesis recently underwent a drastic shift. *Cutibacterium acnes* is a lipophilic bacteria that dominates sebaceous skin sites [3,105]. Each pore is dominated by a single, nearly clonal lineage [105] and individuals have their own unique mix of *C. acnes* strains [3]. Earlier research suggested that increased sebum production and *C. acnes* over-proliferation triggered inflammation, abnormal keratinocyte proliferation, and pore duct obstruction [106]. However, healthy individuals have similar to slightly higher *C. acnes* bioburden [107–110]. Recent works support a dogma where hyper-proliferation of particular *C. acnes* phylotypes (phylotypes IA_1_, IA_2_, 1B_1,_ and IC), reduced *C. acnes* phylotype diversity, and collective skin microbial dysbiosis triggers the cutaneous inflammation underlying acne development [105,108,111,112]. These acne associated phylotypes tend to induce more inflammation, display elevated porphyrin production [113], and exhibit excessive lipase activity [114]. The fatty acids produced by lipase metabolism of sebum subsequently attract neutrophils and promote hyperkeratosis [114].

Mild acne is generally treated with topical benzoyl peroxide and/or a topical retinoid [115]. Topical then oral antibiotics followed by oral isotretinoin is the mainstay for moderate-severe or refractory acne treatment [115]. Concerns for rising antibiotic resistance and isotretinoin's side effects have driven efforts to identify new therapies designed to prevent or reverse the hyperproliferation of *C. acnes* strains and equilibrate the microbiome. One promising avenue is selective augmentation of *S. epidermidis* over *C. acnes* [116,117] through sucrose supplementation [116] or a probiotic containing encapsulated *S. epidermidis* and glycerol [118]. Supplementing the skin microbiome with topical probiotics derived from *Lactobacillus* is another promising avenue [119]. Additional future therapeutics include; i) topical application of anti-microbial peptides [72,120], ii) bacteriophages that strategically infect *C. acnes* [121]*,* and iii) using oral antibiotics to modulate the gut microbiome and indirectly alter the skin microbiome [122–124].

### Homeostasis throughout adulthood

The adult skin microbiome is stable over the span of years [125]. Collectively, established microbial-microbial interaction networks (Table 1), lasting adult skin physiology, and resilient skin immunity maintain balanced adult skin microbial communities. Adult skin microbiomes are dominated by *Cutibacterium, Corynebacterium, Staphylococcus* and *Malassezia* species [4]. Each body site has a unique microenvironment and particular sweat and sebaceous gland density that dictates the prevailing microbial composition [4]. Once adulthood is reached, matured and enduring skin physiology promotes consistent sebum production, sweat composition, and surface pH, which collectively provide reliable body site microenvironments and nutrient pools [126,127]. The immune system also reaches full maturation in our early twenties [128]. Unwavering immune function [129,130] further encourages appropriate, reliable responses to our established commensal microbiome and infectious insults. These intrinsic features enable large portions of microbial communities on the skin to persist, despite daily environmental changes [125]. Microbial community stability is evidenced by the longitudinal fixation of highly abundant species (e.g. *C. acnes)* [125] along with the persistence of several low abundant taxa, which contribute to our unique microbial signature [125,131].

A portion of the taxa within the skin microbiome are also influenced by environmental surroundings. For example, individuals living in Egypt often have a greater abundance of bacteria within the *Pseudomonadota* phyla [132]; individuals in Cameroon have greater *Staphylococcus* and *Micrococcus* [133]*,* those in South Asia tend to have greater abundance of *Corynebacterium* and *Streptococcus* [134]; while individuals in Japan have high abundance of *Cutibacterium* [133]; and Americans and Europeans have greater *Corynebacterium* species abundance [135]. Collectively this illustrates how individuals living in different geographic locations maintain slightly different skin microbial patterns. Individuals living or working in rural communities also contain more soil and agriculture associated microbes within their skin microbiome [136–138]. We also share more microbial taxa with those living in the same residence [131,139,140] and skin microbiomes become more similar the longer individuals cohabitate [141,142]. In short, the people in our lives, local environment, and geography, partially influence our skin microbial balance. The degree to which a portion of your microbiome shifts with environment changes also appears to be person and situation specific [140–142].

### Advancing age

Intriguingly, of the prominent human microbiomes, the skin microbiome is the best predictor of age [143]. With advancing age, distinct skin changes occur including the decline of collagen synthesis, extracellular matrix fragmentation, and a reduction in skin cell regeneration [144]. These changes can manifest as skin wrinkles and more consequently impaired wound healing. Furthermore, these aging related changes shape microbiome composition.

As the skin barrier changes it can lose its ability to retain water, resulting in a compensatory increase in natural moisturizing factor (NFM) production [145]. NMFs both absorb water and can promote bacterial proliferation and adherence to the skin [146]. Subsequently, increased NMFs is associated with greater abundance of numerous taxa, such as *Corynebacterium, Micrococcus, Streptococcus, Anaerococcus* [127], and a reduction in *Cutibacterium* [127,144]. Skin microbial diversity also broadly increases [127,137,144,147]. Decreasing sebocyte area and sebum production after menopause in females correlates with loss of *Cutibacterium* and an increase in *Corynebacterium, Streptococcus, Acinetobacter,* and *Corynebacterium* abundance [127,144,148–150]. In males, sebum secretion declines significantly slower so they maintain greater *Cutibacterium* abundance as they age [137,151].

With age, immune system function also slowly declines [128,130,152,153]. Elderly individuals sustain a low-grade inflammatory state with increased systemic concentrations of pro-inflammatory cytokines [152]. Within the skin Langerhans cells are gradually lost from the epidermis [154]. Cutaneous dendritic cells and the remaining Langerhans also display impaired ability to migrate to lymph nodes and present antigens to T-cells [155,156]. Subsequently, this disrupted antigen presentation along with systemic and local defects in immune signaling ultimately lead to slower immune responses, reduced antimicrobial activity, and impaired wound healing [153,157]. Collectively, impaired immune defenses and increased prevalence of potentially pathogenic bacteria (e.g. beta-hemolytic Streptococci) contribute to the substantial increased risk for skin infection in the elderly and difficulty clearing the infection [153,158,159].

A myriad of dermatologic diseases are associated with advancing age, including dry skin, seborrheic dermatitis, rosacea, disrupted wound healing, and chronic wound infections. Although all these conditions are associated with skin microbiome dysbiosis, specific underlying bacterial and fungal changes remain elusive [18,160,161]. One key exception is seborrheic dermatitis, where recent works find increased abundance of *Malassezia* and *Staphylococcus* species to be potential fungal and bacterial biomarkers [162,163]. In tandem with the numerous works seeking to identify avenues that support a balanced, ‘youthful,' microbiome throughout adulthood and later life [144,150,151], greater research into the active role of the microbiome as we age and in the development of age-associated diseases are needed.

## Conclusion

There is no single definition of a balanced skin microbiome. While there are core skin microbial members, for any individual, the precise microbial composition is dynamic and unique. This dynamic is influenced by the continuous exchange of microbes with the people we are close to and the world around us. The specific community structure in a particular skin microenvironment is also partially determined by local skin physiology, the microbe–immune interface, and complex microbe–microbe interactions. However, on the human microbiome scale, the largest and somewhat predictable shifts in our skin microbial community compositions occur as we, and our skin, age. Future investigations will continue to elucidate the active role of our dynamic skin microbiome across the lifespan, its implications for dermatologic disease risk and overall health, as well as targeted, microbiome centered therapeutic approaches.

## Perspectives

- The skin and its microbiome are primary interfaces with the environment. The balance of this microbial community influences our risk for various diseases and lifelong health.
- There is no single definition of a balanced skin microbiome. At a microenvironment level, balance is dictated by the skin niche along with complex host immune-microbe and microbe–microbe interactions. For any individual this balance is also dynamic, shifting with us as we age and navigate the environments around us.
- Future works in the field of skin microbiology will likely continue to explore i) the active role of our skin microbiome as we age, ii) the complex immune-microbe and microbe–microbe interactions and how they shift over the lifespan, iii) the cutaneous microbiomes of diverse population demographics, iv) the microbiome's influence in protection from and/or disease pathogenesis, and v) the development of novel therapeutic approaches to strategically modulate the microbial balance.

## Abbreviations

3H3MHA: 3-hydroxy-3-methylhexanoic acid
3M3SH: 3-methyl-3-sullanylhexanol
3SH: 3-sulfanylhexanol
BCAA: Branched chain amino acids
CGSC: Cystine-Glycine-S-conjugate
CoNS: Coagulase negative *Staphylococcus* spp
GC: Glutaminyl-conjugate
